# Prediction of Means and Variances of Crosses With Genome-Wide Marker Effects in Barley

**DOI:** 10.3389/fpls.2018.01899

**Published:** 2018-12-21

**Authors:** Tanja Osthushenrich, Matthias Frisch, Carola Zenke-Philippi, Heidi Jaiser, Monika Spiller, László Cselényi, Kerstin Krumnacker, Susanna Boxberger, Doris Kopahnke, Antje Habekuß, Frank Ordon, Eva Herzog

**Affiliations:** ^1^Institute of Agronomy and Plant Breeding II, Justus Liebig University, Gießen, Germany; ^2^Saatzucht Josef Breun GmbH & Co. KG, Herzogenaurach, Germany; ^3^Syngenta Seeds GmbH, Bad Salzuflen, Germany; ^4^W. von Borries-Eckendorf GmbH & Co. KG, Leopoldshöhe, Germany; ^5^Limagrain GmbH, Edemissen, Germany; ^6^Ackermann Saatzucht GmbH & Co. KG, Irlbach, Germany; ^7^Institute for Resistance Research and Stress Tolerance, Julius Kühn-Institute, Quedlinburg, Germany

**Keywords:** cross prediction, genomic prediction, variance prediction, segregation variance, genetic variance

## Abstract

**Background:** The expected genetic variance is an important criterion for the selection of crossing partners which will produce superior combinations of genotypes in their progeny. The advent of molecular markers has opened up new vistas for obtaining precise predictors for the genetic variance of a cross, but fast prediction methods that allow plant breeders to select crossing partners based on already available data from their breeding programs without complicated calculations or simulation of breeding populations are still lacking. The main objective of the present study was to demonstrate the practical applicability of an analytical approach for the selection of superior cross combinations with experimental data from a barley breeding program. We used genome-wide marker effects to predict the yield means and genetic variances of 14 DH families resulting from crosses of four donor lines with five registered elite varieties with the genotypic information of the parental lines. For the validation of the predicted parameters, the analytical approach was extended by the masking variance as a major component of phenotypic variance. The predicted parameters were used to fit normal distribution curves of the phenotypic values and to conduct an Anderson-Darling goodness-of-fit test for the observed phenotypic data of the 14 DH families from the field trial.

**Results:** There was no evidence that the observed phenotypic values deviated from the predicted phenotypic normal distributions in 13 out of 14 crosses. The correlations between the observed and the predicted means and the observed and predicted variances were *r* = 0.95 and *r* = 0.34, respectively. After removing two crosses with downward outliers in the phenotypic data, the correlation between the observed and predicted variances increased to *r* = 0.76. A ranking of the 14 crosses based on the sum of predicted mean and genetic variance identified the 50% best crosses from the field trial correctly.

**Conclusions:** We conclude that the prediction accuracy of the presented approach is sufficiently high to identify superior crosses even with limited phenotypic data. We therefore expect that the analytical approach based on genome-wide marker effects is applicable in a wide range of breeding programs.

## Introduction

Selection gain in breeding programs relies on the selection of suitable crossing partners which will result in derived lines with superior performance. The best cross is not necessarily the cross with the greatest mean performance, but the cross of which the best lines show the highest performance (Zhong and Jannink, 2007). Looking at the criteria which have been suggested to evaluate the potential of a certain cross to generate high-performing progeny, such as the usefulness criterion *U* = μ + *iσ*_*g*_*h* (Schnell and Utz, 1975) or the superior progeny value *s* = μ + *iσ*_*g*_ (Zhong and Jannink, 2007), it becomes clear that the expected genetic variance within a cross is the key factor for identifying the best crosses. Nevertheless, strategies for identifying superior crosses in applied breeding programs have so far mostly relied on pedigree information, mid-parent performance and phenotypic evaluation (Lado et al., 2017). The main reason why the selection of crosses on the basis of their progeny variance has not yet been widely implemented in plant breeding programs was that before the advent of molecular markers there were only limited possibilities of obtaining sufficiently precise predictors for these genetic variances.

In the era of high-throughput genotyping and genomic selection, recent research has focused on obtaining predictors for the genetic variance from genome-wide marker estimates by either simulations (Bernardo, 2014; Lian et al., 2015; Mohammadi et al., 2015) or analytical approaches (Zhong and Jannink, 2007; Bonk et al., 2016; Lehermeier et al., 2017). Versatile analytical methods that allow plant breeders to make a fast selection of superior crossing partners based on already available genotypic and phenotypic data from their breeding programs without the need of reparametrization of estimated marker effects, complicated calculations, or simulation of breeding populations promise to improve the efficiency of breeding programs. In a previous study, we have presented an analytical approach for the prediction of the means and genetic variances of crosses based on maker effects estimated by methods of genomic selection that works for arbitrary mapping functions and mating systems (Osthushenrich et al., 2017). First promising results of cross prediction with analytical approaches were published for simulated populations or multi-parental mapping populations (Bonk et al., 2016; Lado et al., 2017; Lehermeier et al., 2017; Osthushenrich et al., 2017). However, as the design of mapping populations deviates from the design of typical breeding populations, the practical applicability of the analytical approaches in plant breeding populations remains to be demonstrated. To our knowledge, no studies are available which investigate the application of analytical approaches for cross prediction for agronomically important complex quantitative traits with data from actual breeding populations.

The aims of the present study were to apply the analytical formulas for prediction of the means and variances of crosses by Osthushenrich et al. (2017) to a data set from a resistance breeding project in barley, and to investigate the model fit for yield in 14 families of doubled haploid (DH) lines derived from crosses of four pre-breeding lines and five registered commercial elite varieties. Our objective was to investigate the practical relevance and applicability of our analytical approach for the identification of superior cross combinations in plant breeding programs.

## Materials and Methods

### Genetic Material

**Figure 1:**
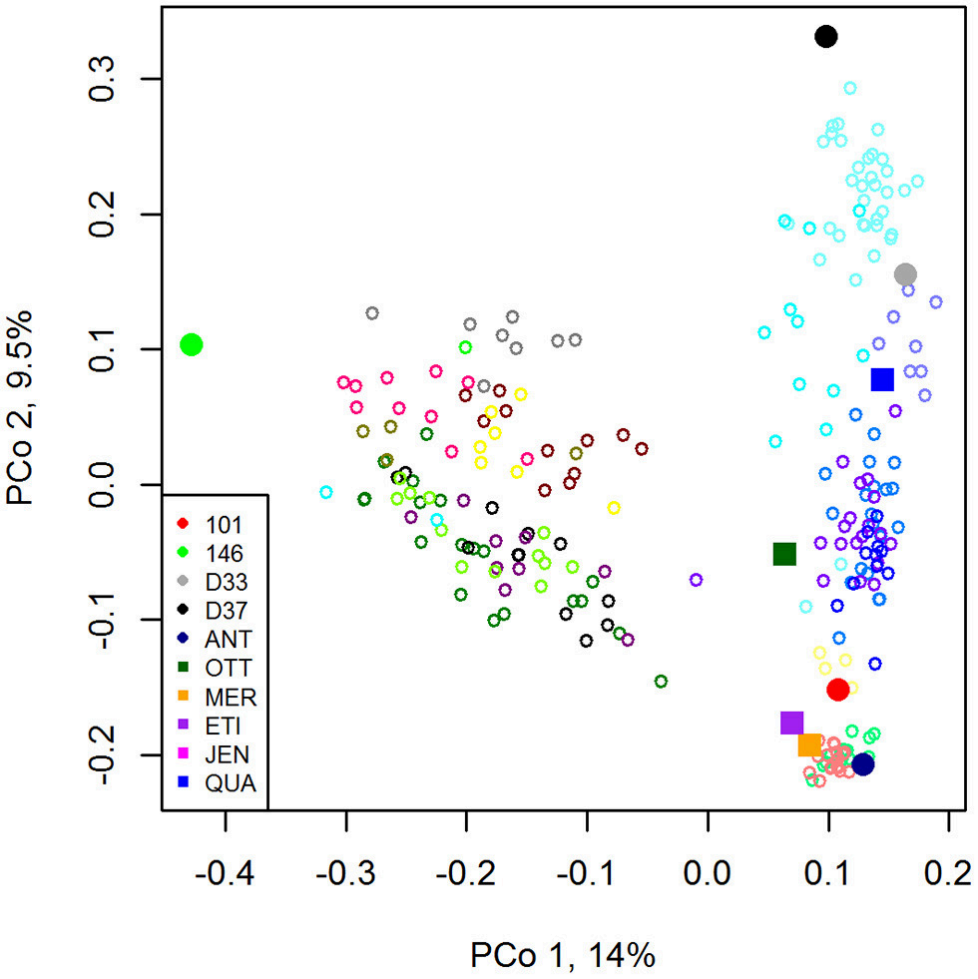
Principal coordinates analysis plot with nine parental lines and 239 emerging DH lines. Parental lines: filled symbols. Circles: resistance donors, Squares: elite lines. DH lines: bordered circles with 19 different colors representing the crosses with viable offspring.

For a resistance breeding project the registered six-row barley varieties Jenny (JEN, Saatzucht Breun), KWS Meridian (MER, KWS Saat SE), Otto (OTT, W. von Borries-Eckendorf), Etincel (ETI, Secobra), and Quadriga (QUA, Secobra) were crossed with the resistance donor lines BAZ 2L101 (101), BAZ 2L146 (146), DH 33 (D33), DH 37 (D37) developed by the Julius Kühn Institute and the registered variety Antonella (ANT, Nordsaat). The resistance donor lines carried resistances to either barley yellow dwarf virus (BYDV; 101, 146), net blotch (*Pyrenophora teres* f. *teres*; D33, D37), or were a registered variety (ANT) carrying resistance to net blotch, powdery mildew (*Blumeria graminis*) and scald (*Rhynchosporium commune*). By crossing each registered elite variety with each donor line, respectively, a 5 × 5 factorial cross was attempted. However, not all crosses were successful and yielded viable offspring (Table 1). Different numbers of F_1_-DH lines were produced from each successful cross, resulting in 250 F_1_-DH lines in total (Table 1). The genetic relationship between parental lines and the emerging DH lines are displayed in a principal coordinate analysis in Figure 1.

**Table 1:** Size n of the families of DH lines derived from the crosses of Parent 1 x Parent 2.

	——————————————————————–		**Parent 1**	——————————————————————–	
**Parent 2**	**101**	**146**	**ANT**	**D33**	**D37**
	——————————————————————–		n	——————————————————————–	
ETI	14	13	18	20	0
JEN	12	7	0	0	0
MER	19	10	16	22	0
OTT	4	10	4	0	13
QUA	1	10	12	8	37

### Field Data

An augmented design with five blocks was used to evaluate all genotypes for yield in one year at five locations in Germany with one replication per location. The field experiment was carried out in Adenstedt (State Niedersachsen, Region Südhannover), Harzhof (State Schleswig-Holstein, Region Ost-Holstein), Irlbach (State Bayern, Region Niederbayern), Lenglern (State Niedersachsen, Region Südniedersachsen), Morgenrot (State Sachsen Anhalt, Region Östliches Harzvorland). The parental lines were used as checks and were replicated five times.

The field data were analyzed with the mixed linear model

$$\begin{array}{l} Yield\;\sim \;\mu \;+\;Genotypes\;+\;Location\;+\;Location:Blocks\;+\;Error \end{array}$$

where the common mean μ and genotypes were treated as fixed factors, whereas blocks, locations, and heteroscedastic model errors were assumed as random. The resulting adjusted entry means for yield for each DH line were used in further calculations.

### Genotypic Data

All 250 resulting DH lines and the ten parental lines were genotyped with the 50 k iSelect Chip (Trait Genetics, Gatersleben). All SNP markers with more than two recorded alleles, more than 10% missing values and a gene diversity of <10% were excluded from the analysis, as well as all individuals with more than 15% missing marker information. As a result, 9,597 SNP markers and 259 genotypes (249 DH lines and 10 parental lines) remained for the analysis.

### Genomic Prediction of Marker Effects

For the prediction of marker effects, we used ridge-regression best linear unbiased prediction (Meuwissen et al., 2001). As training set for the prediction of marker effects we used the complete genotypic and the phenotypic data of the 249 DH lines from the 5 × 5 factorial which remained after data cleaning.

### Prediction of Cross Parameters ${\hat{\mu }}_{\text{g}}$ and ${\hat{\sigma }}_{\text{g}}^{2}$

For the prediction of the expectation ${\hat{\mu }}_{g}$ and the genetic variance ${\hat{\sigma }}_{g}^{2}$ of the crosses we used the analytical approach of Osthushenrich et al. (2017) and the marker effects estimated with RR-BLUP. The required recombination frequencies were derived from a published linkage map (Bayer et al., 2017). We used the genotypes of the ten parental lines to predict ${\hat{\mu }}_{g}$ and ${\hat{\sigma }}_{g}^{2}$ of the resulting DH lines of the validation set.

### Validation Set

For validating the prediction of ${\hat{\mu }}_{g}$ and ${\hat{\sigma }}_{g}^{2}$, we compared the predictions from the formulas with the observed phenotypic values $\bar{x}$ and $s_{p}^{2}$ from the field trial. As validation set, we used the 200 DH lines resulting from the following 14 crosses: 146ETI, 146JEN, 146MER, 146OTT, 146QUA, ANTETI, ANTMER, ANTOTT, ANTQUA, D33ETI, D33MER, D33QUA, D37OTT, D37QUA. The remaining crosses did not result in viable offspring. For line 101, the resulting DH lines from all five crosses had to be excluded from the validation set, as the genotype of the parental line 101 did not match the genotype of the resulting DH lines, meaning that a problem with seed identification of the parental line had at some point occurred during the project. The final validation set thus comprised an unbalanced 5 × 4 factorial of 14 families of 200 DH lines in total (Table 1).

#### Comparison of Predicted ${\hat{\mu }}_{\text{g}}$ and ${\hat{\sigma }}_{\text{g}}^{2}$ and Observed Parameters $\bar{\text{x}}$ and ${\text{s}}_{\text{p}}^{2}$

For comparing the predicted and the observed values from the field trial, we used the yield data of the validation set (Table 1). As the variance of the phenotypic data is defined as $\sigma_{p}^{2}=\sigma_{g}^{2}+\sigma_{m}^{2}$, the approach of Osthushenrich et al. (2017) was extended by an estimate of the distribution of the phenotypic data by adding an estimate $s_{m}^{2}$ of the masking variance $\sigma_{m}^{2}$ to the predicted variance ${\hat{\sigma }}_{g}^{2}$. For this purpose, the masking variance $s_{m}^{2}$ was estimated as the square of the average standard error of the adjusted treatment mean of the mixed models analysis of the field trial (Piepho and Möhring, 2007).

Due to the balanced design of the field trial, the estimated masking variance $s_{m}^{2}$ resulted in the same value of 33.41 dt^2^/ha^2^ for all 14 crosses. An Anderson Darling goodness-of-fit test (Anderson and Darling, 1954) was carried out to test the null hypothesis that the observed yield values of the 14 DH families are a sample from a normal distribution $N\left({\hat{\mu }}_{g},{\hat{\sigma }}_{g}^{2}+s_{m}^{2}\right)$.

### Ranking of Crosses

To validate the identification of superior cross combinations with the analytical approach of Osthushenrich et al. (2017), we created a ranking of crosses based on the criterion ${\hat{\mu }}_{g}+{\hat{\sigma }}_{g}$. This predicted ranking of the crosses was compared to the ranking of crosses based on the best-performing DH line from each cross.

### Software

The statistical analysis of the field data was conducted in R version 3.4.2 (R Core Team, 2017). The estimation of marker effects as well as the prediction of the means and genetic variances of the crosses was conducted in R version 3.4.2 with the software package SelectionTools, which is freely available for download from our homepage^1^. A code and output example is available in **Figure 5**.

## Results

**Figure 2:**
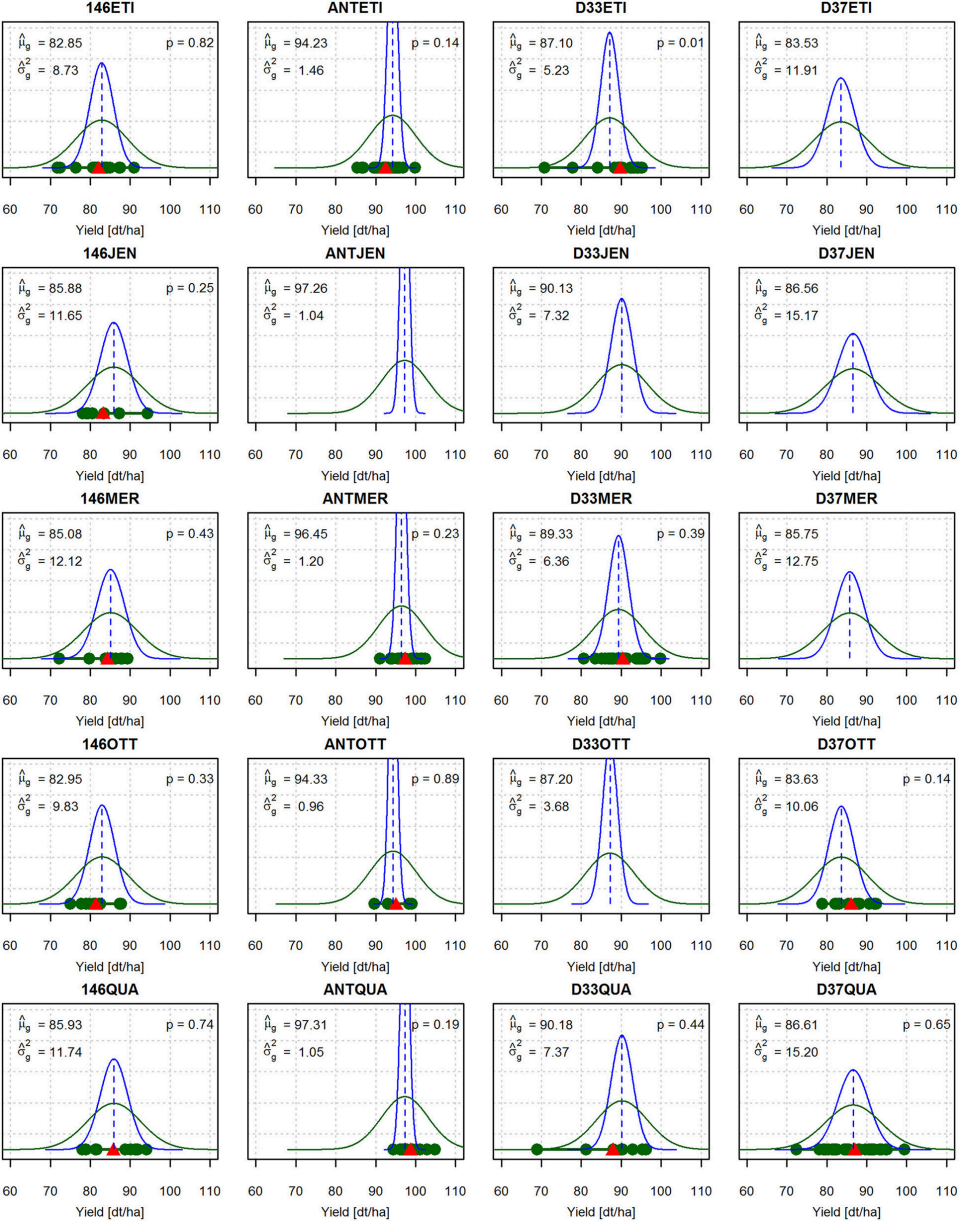
Marker-based predictions of the genetic means ${\hat{\mu }}_{g}$ and variances ${\hat{\sigma }}_{g}^{2}$ of the DH lines derived from all crosses of the complete factorial. The density of a normal distribution with the predicted genetic parameters $N\left({\hat{\mu }}_{g},{\hat{\sigma }}_{g}^{2}\right)$ is depicted in blue. The density of a normal distribution with the predicted phenotypic parameters $N\left({\hat{\mu }}_{g},{\hat{\sigma }}_{g}^{2}+s_{m}^{2}\right)$ is depicted in green, where $s_{m}^{2}$ is the estimated masking variance obtained as the square of the standard error of the adjusted phenotypic means of the field trial. For the crosses for which field data are available, the adjusted treatment means are marked with green dots and the respective family means $\bar{x}$ with red triangles. *p* is the *p*-value of the Anderson-Darling goodness-of-fit test for the null hypothesis that the observed adjusted treatment means are a sample of a normal distribution $N\left({\hat{\mu }}_{g},{\hat{\sigma }}_{g}^{2}+s_{m}^{2}\right)$.

The observed mean yield performance ${\hat{\mu }}_{g}\;$of the crosses ranged from 82.85 dt/ha (146ETI) to 97.31 dt/ha (ANTQUA) (Figure 2). The genetic variances ${\hat{\sigma }}_{g}^{2}\;$ranged from 0.96 dt^2^/ha^2^ (ANTOTT) to 15.20 dt^2^/ha^2^ (D37QUA). The differences between the predicted yield means ${\hat{\mu }}_{g}$ and the genetic variances ${\hat{\sigma }}_{g}^{2}$ were larger between crosses of the same elite variety with different donor lines (columns of Figure 2) than between crosses of the same donor line with different elite varieties (rows of Figure 2). For example, the crosses of the elite variety QUA with four donor lines showed a comparatively large variation of ${\hat{\mu }}_{g}$ and ranged between 85.93 dt/ha and 97.31 dt/ha (last row of Figure 2). The genetic variance ${\hat{\sigma }}_{g}^{2}$ also showed a comparatively large variation and ranged between 1.05 dt^2^/ha^2^ and 15.20 dt^2^/ha^2^. In contrast, for the five crosses with donor line 146, ${\hat{\mu }}_{g}$ for yield ranged only between 82.85 dt/ha and 85.93 dt/ha, and ${\hat{\sigma }}_{g}^{2}$ ranged only between 8.73 dt^2^/ha^2^ and 12.12 dt^2^/ha^2^ (first column of Figure 2). Crosses with donor line ANT, which is a highly resistant elite variety, displayed the overall highest values of ${\hat{\mu }}_{g}$ and the lowest values of ${\hat{\sigma }}_{g}^{2}$ (second column of Figure 2).

The crosses D33ETI and D33QUA showed downward outliers which resulted in high observed phenotypic variances $s_{p}^{2}$ of 36.57 dt^2^/ha^2^ and 80.64 dt^2^/ha^2^ (data not shown, but outliers visible in Figure 2). The phenotypic variances of the other twelve crosses with viable offspring ranged between 9.38 and 36.46 dt^2^/ha^2^ (data not shown). The estimate of the masking variance based on the average standard error from the field data was $s_{m}^{2}=33.41$ dt^2^/ha^2^ and thus was higher than the observed phenotypic variances for ten out of 14 crosses (data not shown).

The Anderson-Darling goodness-of-fit test indicated that there is no evidence to reject the null hypothesis that the observed yield values (Figure 2, green dots) are sampled from a normal distribution $N\left({\hat{\mu }}_{g},{\hat{\sigma }}_{g}^{2}+s_{m}^{2}\right)$ (green curves) in 13 out of 14 crosses. The exception was cross D33ETI which featured downward outliers and a left-skewed sample distribution (*p* = 0.01).

The correlation between the observed yield means $\bar{x}$ (Figure 2, red triangles) and the predicted yield means ${\hat{\mu }}_{g}$ was *r* = 0.95 (data not shown). The correlation between the observed phenotypic variance $s_{p}^{2}$ and the predicted genetic variance ${\hat{\sigma }}_{g}^{2}$ was *r* = 0.34 for all 14 crosses (data not shown). However, when the two crosses D33ETI and D33QUA with downward outliers were removed, this correlation increased to *r* = 0.76 (data not shown).

**Figure 3:**
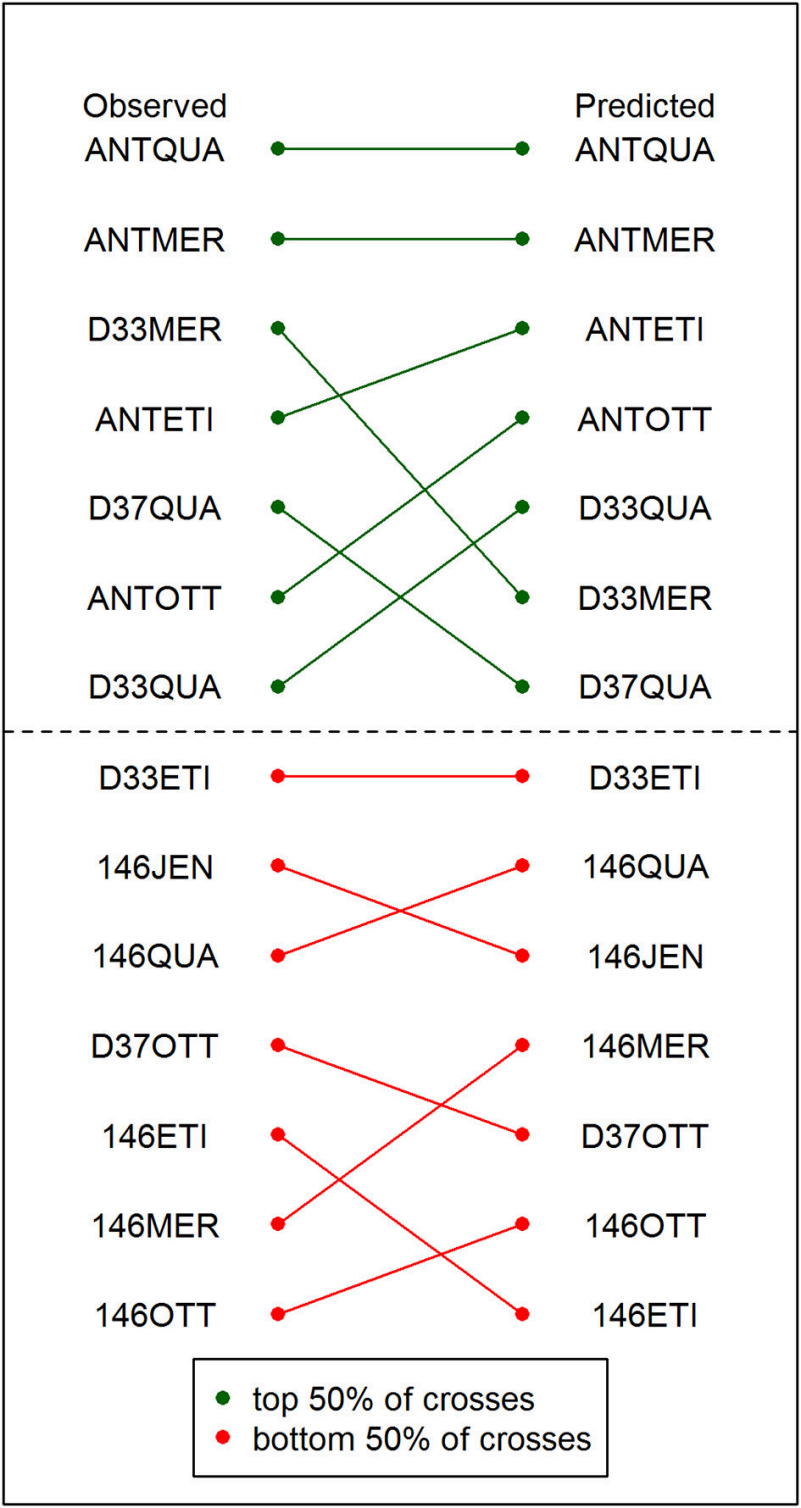
Comparison of the observed and the predicted ranking of crosses. The observed ranking is based on the yields of the best DH lines resulting from each cross in the field trial (**left**). The predicted ranking of crosses is based on the criterion ${\hat{\mu }}_{g}+{\hat{\sigma }}_{g}$ from the predicted cross parameters (**right**). The 50% top-ranked crosses are depicted in green. The 50% bottom-ranked crosses are depicted in red. The green and red lines indicate how the position of the crosses has changed between the observed and the predicted ranking.

A comparison of the ranking of crosses based on the observed yield data of the best resulting DH line from each cross with the ranking of the crosses based on the criterion ${\hat{\mu }}_{g}+{\hat{\sigma }}_{g}$ which relied on the predicted parameters showed that the prediction accuracy was sufficient to correctly identify the 50% best crosses (Figure 3).

**Figure 4:**
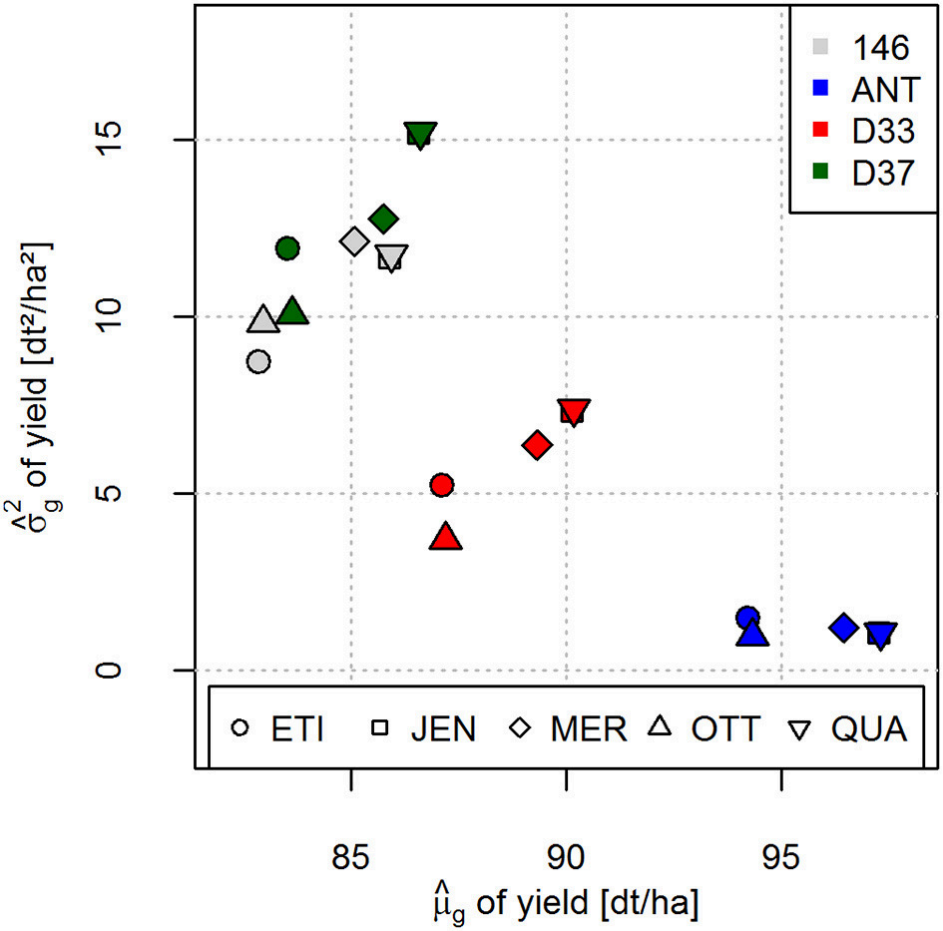
Predicted genetic variances ${\hat{\sigma }}_{g}^{2}$ plotted against the predicted cross means ${\hat{\mu }}_{g}$ for yield for the 20 crosses of four donor and five registered elite varieties. Crosses with the different donor lines are distinguished by different colors (right corner legend). Crosses with different elite varieties are distinguished by different symbols (bottom legend). Specific crosses can be identified by the combination of color and symbol.

A negative covariance existed between ${\hat{\mu }}_{g}$ and ${\hat{\sigma }}_{g}^{2}$ for all crosses (Figure 4). However, when the five potential crossing partners were regarded separately for each donor line, the co-variances between ${\hat{\mu }}_{g}$ and ${\hat{\sigma }}_{g}^{2}$ were positive.

## Discussion

Despite the recent large interest in methods of cross prediction and the selection of promising crossing partners based on marker data in the plant breeding community (Bernardo, 2014; Lian et al., 2015; Mohammadi et al., 2015; Bonk et al., 2016; Han et al., 2017; Lado et al., 2017; Lehermeier et al., 2017), the application of the published analytical approaches was either demonstrated with simulated data sets or in mapping populations which are not comparable in their structure to typical breeding populations. No studies are available to our knowledge in which the applicability of analytical approaches for marker data was tested for relevant traits such as yield in plant breeding data sets. In the present study, we tested if the formulas for variance prediction presented in Osthushenrich et al. (2017) show sufficient precision for the identification of the most promising crossing partners in an ongoing resistance breeding project in barley.

The data set in use in this investigation was not specifically designed for a rigorous validation of the formulas of Osthushenrich et al. (2017). For such a validation study, several parameters would need a different experimental design. We outline these parameters here to show the limits of the present evaluation.

The present study uses a set of intercrossed lines as a training set, and we evaluate the genetic variance in the same data set. Consequently, the results presented here cannot be regarded as an independent validation. Instead, we are rather investigating the fit of the model to the data. If the model does not fit the data in such an analysis, the conclusion can be drawn that the model is not suitable to explain the data. If the model is able to explain the data, however, a considerable overfitting of the model might still be present, because genomic prediction is an *p* > *n* problem where the number of independent variates (*p*, markers) is greater than the number of observations (*n*, lines). This potential overfitting was not quantified by the analyses we present here.

We are using only small numbers of lines per cross. The estimates of the phenotypic variances within each cross are therefore not estimated with a high precision, but instead they have large standard errors and large confidence intervals. In an experiment designed to validate the formulas for variance prediction, larger family sizes would be desirable.

Due to their large standard errors, we decided not to further decompose the per-cross variances into genetic variance and within-cross residual variance. Such an analysis would have the advantage of being able to compare genetic within-cross variances, and in addition would be able to model cross-specific residuals. Nevertheless, the estimation errors of genetic variances are large, even for experiments that were designed specifically for that purpose, and in the present data set we consider the precision of per-cross estimates of genetic variances as too low for drawing valid conclusions. For this reason, we decided to present only the phenotypic per-cross variances, and to compare those with the masking variance estimated across all crosses. This enables an explorative comparison of the magnitudes of the variance components. In a purposely designed experiment, the estimation of per-cross genetic variances and their comparison with the predicted genetic variance would provide not only an explorative comparison but rather would allow more stringent hypothesis testing.

The field trial in our experiment consisted of five replications for each genotype, this resulted in a limited precision of the phenotypic data. As a consequence, the masking variance in our experiment still amounts to considerable size. In a validation experiment carrying out replicated trials in more than five locations and more than one year would result in a smaller masking variance. Ideally, the design of the validation experiment should result in a masking variance that is smaller than the within-family variance. This would allow an effective within-family selection. Further, it would be desirable if the validation experiment was of a size that allowed heteroscedastic error variances for locations or even for the location:cross combinations.

A further issue that is not addressed with our experimental setup is the question of whether random genetic drift or selection during the DH process might have an effect on the estimated variances, this might also be addressed in a validation experiment.

Our motivation to use the present data set in spite of its limitations and in spite of the fact that it was not specifically designed for validation of formulas for variance prediction was, that it actually originates from a practical breeding program. Our argumentation is that the results presented here have a high transferability to applied breeding programs, whereas the results of a pure validation study would have only a limited transferability due to differences in the experimental setup.

The prediction of the yield means ${\hat{\mu }}_{g}$ and genetic variances ${\hat{\sigma }}_{g}^{2}$ of the 14 crosses of five registered elite varieties and four resistance donors for which phenotypic data was available yielded overall plausible results (Figure 2). For example, for the crosses of the elite variety QUA with four donor lines (last row of Figure 2), ${\hat{\mu }}_{g}$ for yield ranged between 85.93 dt/ha and 97.31 dt/ha and ${\hat{\sigma }}_{g}^{2}$ ranged between 1.05 dt^2^/ha^2^ and 15.20 dt^2^/ha^2^. For the five crosses with donor line 146 (first column of Figure 2), ${\hat{\mu }}_{g}$ for yield ranged only between 82.85 dt/ha and 85.93 dt/ha and ${\hat{\sigma }}_{g}^{2}$ ranged between 8.73 dt^2^/ha^2^ and 12.12 dt^2^/ha^2^. Differences between the crosses in ${\hat{\mu }}_{g}$ and ${\hat{\sigma }}_{g}^{2}$ were thus more influenced by donor lines (columns of Figure 2) than by the elite varieties (rows of Figure 2), indicating that the elite varieties contributed little to the genetic variance ${\hat{\sigma }}_{g}^{2}\;$of the crosses and had similar mean performance ${\hat{\mu }}_{g}$. This is also illustrated by the fact that all crosses of elite varieties with donor line ANT, which is also a highly resistant elite variety, had a comparatively high ${\hat{\mu }}_{g}$ and a low ${\hat{\sigma }}_{g}^{2}$ compared to the other crosses. These findings are reflected in the varying spread of the blue normal distribution curves in Figure 2 with $N\left({\hat{\mu }}_{g},{\hat{\sigma }}_{g}^{2}\right)$ for the different crosses. It is also confirmed by the corresponding values for the observed yield means $\bar{x}$ (red triangles) and the observed variances $s_{p}^{2}$ from the field trial (data not shown).

While a direct comparison of ${\hat{\mu }}_{g}$ and $\bar{x}$ from the field trial is straightforward and yielded a correlation of *r* = 0.95 (data not shown), a direct comparison of ${\hat{\sigma }}_{g}^{2}$ predicted from genetic marker effects with the estimated phenotypic variance $s_{p}^{2}$ from the field trials is problematic and less straightforward.

The data set used in the present study comprises field data from only one year, a very limited number of locations and only one replication. In such a small data set, large standard errors are expected for the estimation of the phenotypic variance $s_{p}^{2}$, which result in large confidence intervals. A confidence interval for an observed variance *s*^2^ of a normal distribution is defined as Bronshtein et al. (2003):

$$\begin{array}{l} \frac{\left(n-1\right)s^{2}}{\chi_{\frac{\alpha }{2};\;n-1}^{2}}\leq \sigma^{2}\leq \frac{\left(n-1\right)s^{2}}{\chi_{1-\frac{\alpha }{2};\;n-1}^{2}} \end{array}$$

For example, $s_{p}^{2}$ of the 13 yield values in the field trial for cross 146ETI was 32.63 dt^2^/ha^2^, resulting in a large 0.95 confidence interval of [16.78; 88.91]. From this we can deduce that the point estimator of the phenotypic variance has only limited accuracy. Moreover, marker-based predictions of ${\hat{\sigma }}_{g}^{2}$ are predictions of the genetic variance within a cross, whereas the variance of the true observed values in a field trial is $\sigma_{p}^{2}=\sigma_{g}^{2}+\sigma_{m}^{2}$, where $\sigma_{g}^{2}$ is the genetic variance and $\sigma_{m}^{2}$ is the masking variance due to environmental effects and inaccuracies of the field trial (Piepho and Möhring, 2007). In the present study, the $s_{m}^{2}$ estimated from the field trial was 33.41 dt^2^/ha^2^, while the predicted genetic variances ${\hat{\sigma }}_{g}^{2}$ ranged between 0.96 dt^2^/ha^2^ for the cross ANTOTT to 15.20 dt^2^/ha^2^ for the cross D37QUA. Thus, $s_{m}^{2}$ was in all crosses about 2–30 times higher than ${\hat{\sigma }}_{g}^{2}$, and was consequently the major component of the phenotypic variance ${\hat{\sigma }}_{p}^{2}$.

To account for $\sigma_{m}^{2}$ in our comparison of predicted and observed variances ${\hat{\sigma }}_{g}^{2}$ and $s_{p}^{2}$, we fitted the green normal distribution curve $N\left({\hat{\mu }}_{g},{\hat{\sigma }}_{g}^{2}+s_{m}^{2}\right)$. We conducted an Anderson-Darling goodness-of-fit test to test the hypothesis that the phenotypic yield values of the DH lines from the field trial are samples from these normal distributions (Anderson and Darling, 1954). There was no evidence that this null hypothesis could be rejected for 13 out of 14 crosses (Figure 2). However, when looking at the absolute values of the observed phenotypic variances $s_{p}^{2}$ (data not shown) and the predicted phenotypic variances ${\hat{\sigma }}_{p}^{2}$, our prediction of ${\hat{\sigma }}_{p}^{2}={\hat{\sigma }}_{g}^{2}+s_{m}^{2}$ tended to overestimate the observed variance $s_{p}^{2}$ of the phenotypic values.

This overestimation could be expected, as precise field trials to assess the yield are only carried out for a limited number of pre-selected individuals, while the analytical approach yields estimates for infinite unselected population sizes. Moreover, the crosses D33ETI and D33QUA featured downward outliers that might have inflated the average standard error for the adjusted treatment means and consequently the derived masking variance $s_{m}^{2}$. Under the assumption that the masking variance $\sigma_{m}^{2}$ is constant for all crosses, the correlation *r* between the predicted genetic variance ${\hat{\sigma }}_{g}^{2}$ and the observed phenotypic variance $s_{p}^{2}$ gives an idea how valid the predictions for the evaluation of suitable crossing partners are. This correlation was *r* = 0.34 for all 14 crosses (data not shown). However, this was also mainly due to the crosses D33ETI and D33QUA, which each displayed outliers in the form of two very low yield values (Figure 2), resulting in high observed variances $s_{p}^{2}$ of the phenotypic values. Excluding these two crosses, the correlation increased to *r* = 0.76 (data not shown). From these findings, we draw two conclusions. First, low correlations between the predicted genetic variances ${\hat{\sigma }}_{g}^{2}$ and the observed phenotypic variances $s_{p}^{2}$ can be caused by outliers in the field trial which result in overestimated phenotypic variances. They do not necessarily mean that the prediction approach in itself is faulty or inaccurate. Rather, accurate field trials are of major importance not only for estimating marker effects and cross prediction, but also for the plausible validation of cross prediction. The evaluation of the accuracy of cross prediction should therefore comprise a careful monitoring of the field data. Estimates of the phenotypic variance $s_{p}^{2}$ from samples with outliers should be treated with caution. Second, the results shown in Figure 2 indicate that our predictions of ${\hat{\sigma }}_{g}^{2}$ overall yielded reasonable results in light of the limitations of the available phenotypic data.

Despite the fact that the predicted genetic variances ${\hat{\sigma }}_{g}^{2}$ are difficult to validate with phenotypic data from breeding programs, they can still improve the efficiency of breeding programs with respect to long-term response to selection and efficient use of the limited plot number for field trials. Even for lower correlations between ${\hat{\sigma }}_{g}^{2}$ and $s_{p}^{2}$ it is reasonable to focus on crosses with high predicted genetic variance in order to maintain genetic diversity and long-term response to selection, given that reliable phenotypic and genotypic data is available for predicting marker effects.

More importantly, we argue that the main application of cross prediction in practical breeding programs is not so much to provide 100 percent accurate predictions of ${\hat{\mu }}_{g}$ and ${\hat{\sigma }}_{g}^{2}$ but to allow the breeder to identify a certain fraction of promising crosses from the complete list of potential crosses in order to use the limited number of field plots efficiently. We compared the ranking of the crosses based on the criterion ${\hat{\mu }}_{g}+{\hat{\sigma }}_{g}$ to the ranking of the crosses based on the yield data of the best resulting DH line from each cross (Figure 3). In this comparison, all seven top-ranked crosses were identified correctly with the predicted parameters, allowing the breeder to efficiently narrow down the number of lines which have to be evaluated in costly field trials by 50% without reduction in selection gain.

It has been postulated that a negative covariance exists between ${\hat{\mu }}_{g}$ and ${\hat{\sigma }}_{g}^{2}$ (Zhong and Jannink, 2007). This suggestion is very reasonable, as elite varieties which are fixed at many loci for superior alleles will result in crosses with high ${\hat{\mu }}_{g}$ and low ${\hat{\sigma }}_{g}^{2}$. This negative covariance is also observed in our data set if ${\hat{\mu }}_{g}$ is plotted against ${\hat{\sigma }}_{g}^{2}$ (Figure 4). For example, the ANT crosses can be considered as crosses between two elite varieties and consequently have a comparatively high ${\hat{\mu }}_{g}$ and low ${\hat{\sigma }}_{g}^{2}$ compared to the other crosses. In our data set, in line with the suggestions of Zhong and Jannink (2007), genetic variances ${\hat{\sigma }}_{g}^{2}$ were more influenced by donor lines (columns of Figure 2) than by the elite varieties (rows of Figure 2), indicating that the elite varieties contributed little to the genetic variances ${\hat{\sigma }}_{g}^{2}$ of the crosses. Crosses of elite varieties with donor lines 146, D33 and D37 which are pre-breeding lines with overall lower agronomic performance have lower ${\hat{\mu }}_{g}$ and higher ${\hat{\sigma }}_{g}^{2}$ in comparison to the ANT crosses.

Thus, we observed that the negative covariance between ${\hat{\mu }}_{g}$ and ${\hat{\sigma }}_{g}^{2}$ of the crosses is mainly due to the different level of breeding intensity and selection that the donor lines have been subjected to (Figure 4). If the crosses of donor lines are regarded separately, as indicated by the different colors in Figure 4, a positive covariance existed between ${\hat{\mu }}_{g}$ and ${\hat{\sigma }}_{g}^{2}$. We therefore conclude that for many scenarios, for example if a specific donor line carrying desired resistance genes has to be used for trait introgression into the breeding pool, prediction of the genetic variance ${\hat{\sigma }}_{g}^{2}$ allows the breeder to identify the best crossing partner for this donor line from a set of different elite varieties. In addition, these predictions can also be used for improved resource allocation by investing more resources in terms of number of progeny into crosses with higher predicted genetic variance ${\hat{\sigma }}_{g}^{2}$. We plan further investigations in this area.

**Figure 5:**
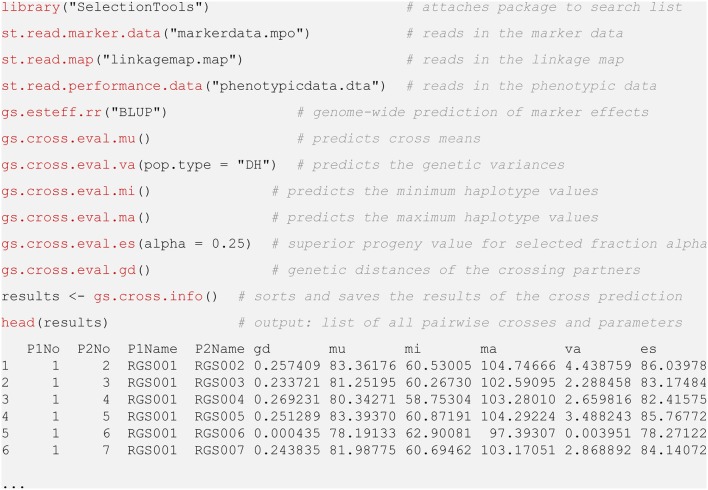
Demonstration of R Code used for cross prediction with package SelectionTools.

In order to provide breeders with a fast and easy-to-use tool to implement the presented approach in their breeding pipelines, routines for data pre-processing, estimation of marker effects and cross prediction with the formulas of Osthushenrich et al. (2017) have been included in the software package SelectionTools. SelectionTools allows breeders to make use of the advantages of cross prediction in a convenient way without the need of comprehensive mathematical and programming skills. With standard data formats, the presented approach can be reproduced with only a few lines of R code (Figure 5).

## Conclusion

The analytical approach of Osthushenrich et al. (2017) yields plausible cross predictions which allow breeders to establish a ranking of potential crosses and identify a superior fraction of crosses for field evaluation. The approach is versatile and can be used for arbitrary mating systems. A major advantage of the presented approach is that it can be directly and easily used with marker effects from genome-wide prediction without time-consuming additional calculations or simulations. The prediction accuracy of means and variances is sufficiently high for practical application to derive meaningful predictions even with limited phenotypic data. We therefore expect that the formulas are applicable in a wide range of breeding programs.

## Availability of Data and Material

The datasets generated and/or analyzed during the current study are not publicly available due to the confidential genotypic data of the donor lines from an ongoing research project but are available from the corresponding author on reasonable request.

## Author Contributions

HJ, MS, LC, KK, SB, AH and DK developed the genetic materials, conducted the field and greenhouse experiments. CZP and MF planned the field experiments and analyzed the field data. TO analyzed the genotypic data. TO and EH wrote the manuscript. MF, FO, and EH directed the project, contributed to the analyses and manuscript revisions. All authors proof-read the draft and approved the final manuscript.

### Conflict of Interest Statement

The authors declare that the research was conducted in the absence of any commercial or financial relationships that could be construed as a potential conflict of interest.

## Footnotes

^1^www.uni-giessen.de/population-genetics Homepage of the Department of Biometry and Population Genetics, Institute for Agronomy and Plant Breeding II, Justus Liebig University Giessen. www.uni-giessen.de/population-genetics. Accessed 21 February 2018.1514.

## Abbreviations

DH: doubled haploid.
